# Overcommitment, Work-Related Behavior, and Cognitive and Emotional Irritation in Veterinarians: A Comparison of Different Veterinary Working Fields

**DOI:** 10.3390/healthcare12151514

**Published:** 2024-07-30

**Authors:** Beatrice Thielmann, Robert Pohl, Irina Böckelmann

**Affiliations:** Institute of Occupational Medicine, Otto von Guericke University Magdeburg, Medical Faculty, Leipziger Str. 44, 39120 Magdeburg, Germany; robert.pohl@med.ovgu.de (R.P.); irina.boeckelmann@med.ovgu.de (I.B.)

**Keywords:** mental health, veterinary professionals, occupational stress, work-related behavior, cognitive irritation, emotional irritation, overcommitment, AVEM patterns, veterinary specialties, risk assessment

## Abstract

Mental health is a serious problem among veterinarians. The aim of this study was to analyze work-related behaviors and experience (AVEM), overcommitment (OC), and cognitive and emotional irritation (IS) in different veterinary working fields. The survey included 724 German veterinarians (average age 41.0 ± 9.72 years). Validated questionnaires were used to assess overcommitment, work-related behavior and experience patterns (health-promoting pattern G or S; health-hazardous risk pattern A or B), and irritation in several working fields. A correlation analysis and a multivariate test were performed. Increased OC was observed in 35.8% of veterinarians (mixed animals vs. inspectors, *p* = 0.042; small vs. mixed animals, *p* = 0.001). A total of 66% of veterinarians exhibited AVEM risk pattern A or B. There was no significant association of AVEM risk patterns and veterinary specialty. Only the AVEM dimension “tendency toward resignation in the face of failure” differed among working fields (*p* = 0.04). Regardless of direct animal contact, German veterinarians showed increased psychological stress. Inadequate compensation and prolonged stress are significant factors that can lead to burnout or depression. These risks should be considered in the context of occupational healthcare.

## 1. Introduction

The workloads of veterinarians are heavy and vary in psychological, physical, and organizational factors [1,2,3,4,5]. A systematic review showed a high prevalence of psychological stressors in veterinary practice [3]. Subjective stress because of workload was more pronounced in women than in men. Veterinarians face unique stressors that can lead to higher levels of stress compared to other similar professions, such as human medical professionals. The unique nature of their work and the environments in which they work contribute to this increased stress. Following a comparison of stressors faced by veterinarians and human medical professionals, and why stress may be more pronounced in veterinarians, the following was determined: Expected to be generalists, veterinarians treat a wide range of species, requiring broad knowledge and expertise. Human medics typically specialize in a particular area of medicine, allowing for focused expertise. These subspecialties are not common in veterinary medicine. Veterinarians often deal with emotionally charged pet owners who may struggle to pay for treatment, but still expect optimal care. The corresponding counterpart to the health insurance for “human patients”, which is common in the German healthcare system, is missing for “animal patients”. This creates a difficult balance between medical ethics and financial considerations.

Among veterinarians, particularly relevant stressors include long working hours and ethical decision-making, e.g., in the context of euthanasia [3,6,7]. Human medicine is concerned with care at the end of life; euthanasia is less common and often involves palliative care rather than a decision to end life. Another review highlighted reasons for distress, such as workload, financial problems (lower income compared to human medicine specialists) [6], debt accrued while pursuing a degree (differed in each country), difficult client interactions, unexpected patient outcomes, and fear of client complaints or of making mistakes [8,9]. Human–human interactions are the main stressor for veterinarians [10]. A study of German veterinarians demonstrate that a good working atmosphere is the most crucial job characteristic for veterinary practitioners, significantly influencing work satisfaction, particularly among employed practitioners [11].

The results of a study among German veterinarians indicated that 98.7% of the participating veterinarians observed a significant rise in pet owners’ self-education behavior over the past few years, which has noticeably impacted interactions during veterinary appointments [12]. Furthermore, threatening situations and work–life imbalance were identified as other causes of stress [13].

Veterinarians’ stress levels vary greatly depending on their area of practice [14,15,16]. Clinical practice, especially in small and large animal practices, tends to have higher stress levels due to client interactions, physical demands, and emergency services [17,18]. Working with large or mixed animals was a predictor of impaired health [19,20,21].

A more balanced perspective is valuable. The introduction provides a negative view of veterinary work, which is unfortunately widespread. It is important to recognize that the longstanding focus on problems has, of course, found problems. Many veterinarians enjoy and find value in their work, yet there is relatively little research into the strengths and benefits of veterinary work.

### 1.1. Veterinarians’ Level of Stress and Theoretical Models of Stress

Overall, the well-being of veterinary professionals is lower than of the general population. A higher percentage of U.S. veterinarians (9.1%) reported poor well-being than that of the U.S. general population (7.3%) [22]. Risks of burnout, anxiety and depressive disorders, and suicide attempts and fatal suicide are high among veterinarians [1,14,17] and somewhat higher than in the general population and in other occupational groups [3,9,15]. The study by Schwerdtfeger et al. shows that German veterinarians have an increased risk of depression (27.78%), suicidal ideation (19.2%) and suicide risk (32.11%) compared with the general population in Germany (accordingly 3.99%, 5.7%, and 6.62%) [23]. A second survey of the veterinary profession in Europe (more than 13,000 respondents), further showed that veterinarians are exposed to high levels of stress and that many veterinarians had to take two weeks off due to mental health issues [24]. The break must also be considered on a country-by-country basis. Russia (78% of veterinarians) and North Macedonia (63%) led the way, with most European Union countries (26% total) in the middle of the field, and Switzerland (12%) and Germany (12%) at the bottom [24].

It is known that the level of stress depends on intra- and interindividual conditions and coping strategies as well as the personality traits of each person [25]. Well-known theoretical models of the relationships of job demands and job-related stressors with stress suggest that stress occurs when there is an imbalance between job demands and perceived ability to cope. Such theoretical models include the stress–strain concept, developed by Rohmert and Rutenfranz, and occupational gratification crises, developed by Siegrist [26,27]. Overcommitment (OC) is a concept within the effort–reward imbalance (ERI) model developed by Siegrist [27]. The ERI model is a framework that helps explain work-related stress and its impact on health. According to the model, stress occurs when there is an imbalance between the effort put into work and the rewards received in return. Rewards can include pay, career opportunities, job security, and appreciation/recognition. According to Siegrist, overcommitment is the tendency to engage in excessive professional commitments. OC is an intrinsic personal trait that predisposes individuals to excessive work-related effort.

The Work-related Behavior and Experience Patterns (AVEM) model assesses coping strategies and stress responses in the work context [28,29]. AVEM can be viewed as multidimensional patterns that reflect an individual’s coping strategies and stress responses in a work context: healthy patterns G and S, risky patterns A and B [30]. These patterns may be influenced by, but should be distinguished from, underlying personality traits [28,29].

A noticeable imbalance between effort and reward leads to a “gratification crisis”, which is followed by pronounced stress and mental and physical illnesses [27]. Studies have shown an increased risk of psychological symptoms, such as depression, emotional exhaustion, burnout, cardiovascular disease, and anxiety, with higher values of overcommitment [31,32,33,34,35]. Overcommitment is considered a risk factor for mental and physical health problems, especially when combined with an imbalance between effort and reward. Similarly, health-promoting or risky work-related behaviors and experiences can promote or threaten health, respectively [30]. Risky work-related behavior and experiences have been shown to lead to emotional dysfunction, cardiovascular complaints, vegetative complaints, sleep disturbances, exhaustion and impaired memory and concentration. In contrast, health-promoting work-related behaviors and experiences led to the fewest health complaints [30].

### 1.2. Identification of Subclinical Stress Symptoms in Occupational Medicine

Occupational medicine, along with health surveillance for health promotion and prevention, plays a critical role in identifying the early stages of stress before it develops into an acute or chronic condition. Questionnaires, such as the Irritation Scale (IS), are effective in detecting subclinical stress symptoms such as cognitive and emotional irritation [36]. This involves the health-friendly design of work and organization, i.e., the economic assessment of employees’ state of health.

Irritation is hardly described in the literature, but offers the advantage described above in the survey of the health status of employees: compensable stress states.

### 1.3. Derivation of the Objective and Working Hypotheses

The reason for conducting this study is the reported high levels of stress in the veterinary profession, with a lack of comprehensive research, particularly in relation to preventative measures for occupational health. By focusing on simple assessment tools, such as the measurement of irritation, this study aims to provide actionable insights. As already mentioned, irritation provides indications of stress reactions to workloads which, although not pathological, indicate a need for intervention. AVEM offers further opportunities to introduce targeted preventive measures. This can help to identify stress-related problems at an early stage and develop targeted prevention strategies to improve the well-being of veterinarians.

The aim of this study was to determine associations among work-related behavior and experience, overcommitment, and cognitive and emotional irritation among veterinarians. In addition, these resource- and stress-related data were compared among veterinary working fields. It was hypothesized that veterinarians in direct contact with animals (small animals, large animals) will show more of the following than veterinarians in other working areas (e.g., laboratory, public administration):

- higher overcommitment;
- more risky work behaviors patterns (A and B);
- higher cognitive irritation and emotional irritation.

## 2. Materials and Methods

From 1 July 2021 to 31 February 2023, a nationwide online survey of German veterinarians was conducted. The extended survey period allows veterinarians who have been ill or absent for a longer period of time to participate in the study. In addition, conducting a nationwide survey helps to avoid selection bias. The online survey used the platform soSci (version 3.2.03-I, Munich, Germany). This work was supported by the Professional Association for Health Services and Welfare Care (BGW), which operates in Germany. The study was approved by the Ethics Committee of Otto-von-Guericke University Magdeburg (No. 91/21). The complete protocol of the project “Causes and consequences of psychological stress in the working life and emergency services of veterinary professionals in the Federal Republic of Germany” has been published [37] and can be viewed in the German Register of Clinical Studies under the registration number DRKS00026106. Participants were recruited by disseminating information about the study through the Federal Veterinary Chamber, the State Veterinary Chambers on their respective websites, and the German Social Accident Insurance Institution for the health and welfare services (BGW—Berufsgenossenschaft für Gesundheitsdienst und Wohlfahrtspflege). The link to the survey was posted on the official websites of the various State Veterinary Chambers. Preliminary letters were drafted and sent to the relevant authorities to obtain formal support. In addition, targeted requests were made to veterinary professional associations, clubs, and other relevant organizations to secure their assistance in recruiting potential participants. These institutions were actively contacted to inform their members about the study and to encourage participation. In addition, a close collaboration was established with the Veterinary Chamber of Saxony-Anhalt to provide direct access to veterinarians in this region. This partnership aimed to increase participation through targeted communication and cooperation. Additional information was published in the German Veterinary Journal (issue 09/2021) and via social media channels. The STROBE checklist for cross-sectional studies was observed [38]. Participants were able to access the survey via a QR code. The response rate was 3.0%. Taking into account that 33,326 veterinarians were employed or self-employed in Germany in 2022 [39], the representativeness for German veterinarians can be classified as low. Sample size was calculated using the G*Power program (version 3.1.9.7, Heinrich-Heine-University Düsseldorf, Düsseldorf, Germany). A total sample of at least *n* = 232 was calculated for the quantitative survey.

### 2.1. Sample

The overall project included 999 veterinarians who completed the relevant questionnaires. Subjects with missing data in the questionnaires presented here were excluded. Therefore, the present study included data from 724 veterinarians with a mean age of 41.9 years ± 10.17 SD (23–79 years).

The inclusion criteria for veterinarians were as follows: worked in the Federal Republic of Germany and had over one year of working experience in the field of veterinary medicine.

The veterinarians worked in small animal medicine, large animal medicine, laboratory research, and public administration as well as “other”. Since the number of respondents who selected “other” was less than 5, this group was excluded from the statistical analyses. Participation in the study was voluntary and was assumed by completing the online questionnaire. Non-physician personnel in veterinary practices or trainee veterinarians were excluded.

### 2.2. Methods

The following data were evaluated:

- demographic data (e.g., age, sex);
- main working field, in accordance with the Saxony-Anhalt Chamber of Veterinarians,
  ○ with direct contact with animals such as small animals (e.g., pets, birds), large animals (e.g., farm animals, horses), or both small and large animals;
  ○ without direct contact with animals, such as veterinarians working in laboratories or working in public administration (e.g., animal welfare office, veterinary officers, authority).
- The variables of occupational–psychological measures were as follows:
  ○ overcommitment (OC) [27];
  ○ work-related behavior and experience patterns (AVEM) [28,29];
  ○ irritation (measured on the Irritation Scale; IS) [36].

The questionnaires used in this study were developed and validated with a German population. The occupational–psychological questionnaires are described in more detail below.

#### 2.2.1. Overcommitment (OC)

Overcommitment, according to Siegrist et al. [27], involves excessive occupational expenditure. OC is part of the questionnaire for the measuring of occupational gratification crises (ERI) according to Siegrist et al. [27]. OC can be considered separately as an intrinsic component in ERI model. An OC can arise, for example, if the work requirements exceed the employee’s competencies, or if the employee does not meet the requirements profile or develops too strong a willingness to perform for the job. A high need for recognition or the fear of failure at work, an increased competitive drive, an increased feeling of time pressure or the inability to distance oneself from the job performance demands promote OC.

The related scale has 6 items scored on a 4-point scale: strongly disagree (1) to strongly agree (4). After the pole reversal of the 3rd item, all marked answers of questions 1 to 6 are summed up. The total score ranges between 6 and 24 points. A value ≥ 18 (75th percentile of our results) is used as the threshold for overcommitment. A high OC point score indicates a high tendency for the participant to feel overwhelmed. A value of ≥18 corresponds to a critical OC level. The OC level is dichotomous, involving a normal or high expenditure tendency. For the individual scales, the Cronbach’s alpha values are between 0.637 and 0.801. If all items remain in the scale, we have a Cronbach’s alpha of 0.709 (acceptable according to Blanz [40]).

#### 2.2.2. Work-Related Behavior and Experience Patterns

**Figure 1:**
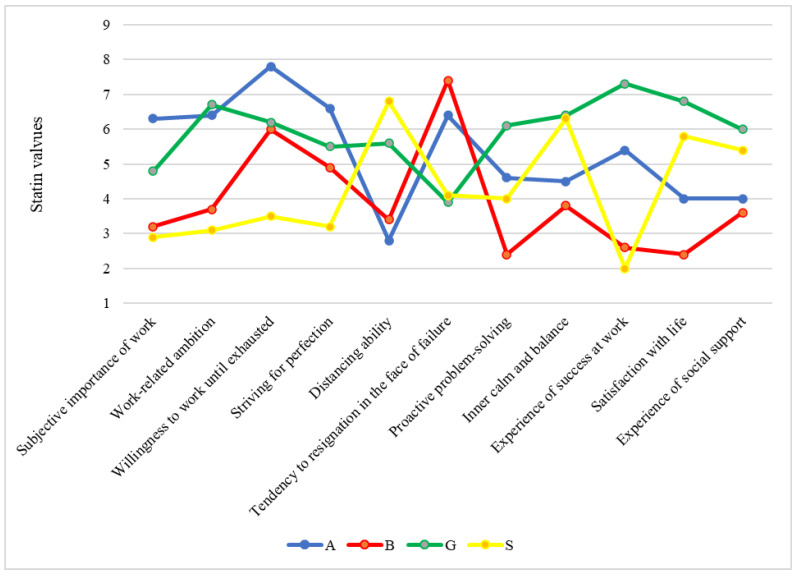
Expressions of AVEM dimensions of the four AVEM pattern of all veterinarians (Notes: normal range is from 4 to 6 stanine values).

The Work-related Behavior and Experience Patterns (AVEM; in German Arbeitsbezogene Verhaltens- und Erlebensmuster) questionnaire developed by Schaarschmidt and Fischer [28,29], collects information on the aspects of coping with work and coping with occupational requirements. The standard version, which has 66 items scored on a 5-point scale (“…completely true”, “…mostly true”, “…partly/partly true”, “…mostly not”, and “does not apply at all”), was used. The AVEM evaluates the following eleven dimensions of work-related behavior and experience: 1. Subjective importance of work, 2. Work-related ambition, 3. Willingness to work until exhausted, 4. Striving for perfection, 5. Distancing ability, 6. Tendency toward resignation in the face of failure, 7. Proactive problem solving, 8. Inner calm and balance, 9. Experience of success at work, 10. Satisfaction with life, and 11. Experience of social support. These eleven dimensions can be grouped into three areas: “engagement with work” (dimensions 1–5), “resilience in dealing with the everyday stresses of work” (dimensions 5 (again) and 6–8) and “emotions associated with work and with life in general” (dimensions 9–11). Depending on the scores in the three areas or eleven dimensions, individuals can be assigned to four different patterns of work-related experience and behavior determined via cluster analysis: two risk patterns regarding health (patterns A and B) and two health-promoting patterns (G and S). Figure 1 shows the variations in the dimensions of work-related behavior and experience across each AVEM pattern. The results of our sample are taken as the basis for these figures. The test results are mapped into a nine-point scale. Such a stanine scale then corresponds to a scale modified at the edges, with a mean of 5 and a standard deviation of 2 points. Therefore, a normal expression lies in the range of 4 to 6 stanine values. A below-average expression lies in the range of 1 to 3 and an above-average expression from 7 to 9.

Typical characteristics for each AVEM pattern are:

- *Health-promoting pattern G:* A good attitude towards work with a positive effect on health and great importance of engagement in work, high resistance to stress, health “distancing ability”. Strongest values: “work-related ambition”, “proactive problem-solving”, “inner calm and balance”, moderately to slightly elevated values: “subjective importance of work”, “willingness to work until exhausted”, “striving for perfection”. Low values: “Tendency to resignation in the face of failure”.
- *Health-promoting pattern S*: Lowest values: work engagement and well-developed resilience at work. Strongest: “distancing ability”. Low values “tendency to resign in the face of failure”, “experience of success in the job”. High value: “life satisfaction”.
- *Health-hazardous/risk pattern A*: Very strong engagement, high performance pressure, reduced resistance to stress, high effort, no positive emotions. Highest expressions: “subjective importance of work”, “willingness to work until exhaustion”, “striving for perfection”. Relatively high value: “tendency to resignation in the face of failure”. Lowest value: “distancing ability”. Low value: “Inner calm and balance”.
- *Health-hazardous/risk pattern B:* Low expression of engagement, loss of motivation, reduced resilience, negative feelings (reminiscent of a burnout syndrome), “distancing ability”. Predominant value: “resignation tendency in face of failure”. Transitions from risk pattern A to B are possible through random, frequent change from pattern S to risk pattern B.

Individuals were categorized using the Vienna Test System (Schuhfried GmbH, Mödling, Austria) into the different AVEM pattern among the expression of the AVEM dimensions. Schaarschmidt and Fischer [29] differed between pure patterns (with a match >95% to one pattern), accentuated patterns (with matches between >80% and ≤95% in one pattern), and tendential patterns (matches ≥60 to ≤80% in one pattern, with no other pattern having ≥30% match).

Pattern combinations were not considered because they are not described in the manual. For the individual dimensions, the Cronbach’s alpha values range from 0.399 to 0.650 (from unacceptable to questionable according to Blanz [40]). If all items remain in the scale, we have a Cronbach’s alpha of 0.538 (poor/low).

#### 2.2.3. Irritation Scale (IS)

**Figure 2:**
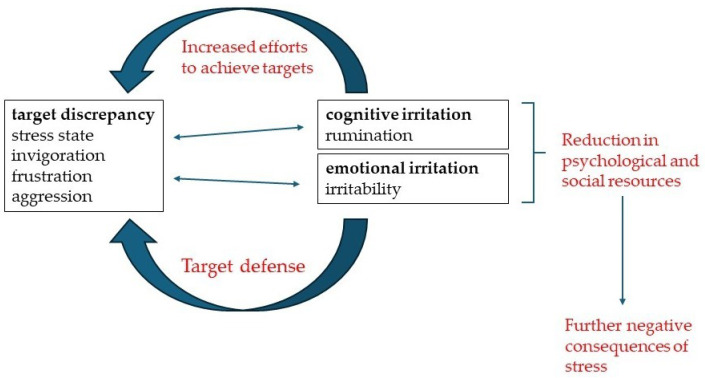
Explanatory approaches of the Irritation Scale; own presentation.

Irritation is a state of psychological impairment between mental fatigue and mental illness [36]. The Irritation Scale measures the cognitive and emotional components of work-related irritation. In such cases, irritation presents a state between mental fatigue and mental illness (i.e., the subclinical state), without the possibility of a diagnosis [36]. Mental fatigue may be so pronounced that a normal recovery period (over a weekend) is insufficient to recover. Cognitive irritation is characterized by persistent work-related rumination, while emotional irritation includes persistent feelings of frustration and anger. A night’s rest distinguishes mental fatigue from irritation: while mental fatigue usually disappears after rest, irritation tends to persist. Chronic irritation is not itself a mental illness, but it may indicate or predict potential mental health problems. The IS construct is shown in Figure 2.

The Irritation Scale consists of eight items that are rated on a seven-point Likert scale (“…strongly disagree”, “…mostly disagree”, “…slightly agree”, “…moderately agree”, “…somewhat agree”, “…mostly agree” and “…almost completely agree”)…

It includes the following:

- *Cognitive irritation subscale:* Characterized by persistent rumination about work problems. Individuals with high cognitive irritation often find themselves thinking about work issues outside of work hours, believing that these repetitive thoughts will help them achieve their goals. For example, they may say, “I have to think about work at home. However, this rumination is counterproductive because it often leads to a “paralysis of action”, where preoccupation with existing problems prevents them from effectively tackling new tasks [36].
- *Emotional irritation subscale:* Reflects verbally aggressive tendencies and irritability, resulting in grumpy reactions or frustration. This type of irritability involves emotional responses to work-related stressors that interfere with goal attainment. Emotional irritation is considered a defensive response to obstacles that impede progress [36].
- *IS total score:* The IS includes eight items and a 7-point response scale from “strongly disagree” to “strongly agree”. The values of the eight items are added up. High values reflect a high degree of irritation. Table 1 shows the interpretation of the score.

Cronbach’s alpha, a measure of internal consistency, was 0.935 for cognitive irritation (excellent according to Blanz [40]), 0.758 for emotional irritation (acceptable), and 0.729 for the total score (acceptable). If all items were included, we obtained a Cronbach’s alpha value of 0.887 (good/high).

### 2.3. Statistical Analysis

SPSS was used for the statistical analyses (version 28.0.0.0, IBM). The descriptive statistics included mean, standard deviation, median, minimum, maximum, and a 95% confidence interval. An evaluation of the data distributions for normality revealed non-normal distributions. The significance level α was set at 5%. The chi-square test or Fisher exact test was used to compare data between sexes and to compare AVEM patterns and OC classifications among veterinary working fields. The main analysis compared veterinary working fields.

Group comparisons of age, IS subscale scores (emotional and cognitive) and IS total scores, overcommitment, and AVEM dimensions were performed with the Kruskal–Wallis test (KW). If significant, pairwise comparisons were made with Bonferroni correction applied. Finally, Spearman correlation analysis was performed. Following Akoglu, Spearman’s rho was classified as follows: weak correlation (0.100 to 0.399), moderate correlation (0.400 to 0.699), or strong correlation (≥0.700) [41]. Finally, an analysis of possible predictors was performed as part of a multivariate test with a test for between-subjects effects. Sex, age, OC score, and working group were adjusted for. The interpretation was also carried out according to the following scheme: η^2^ < 0.06 (weak effect), η^2^ = 0.06 to 0.14 (moderate effect), η^2^ > 0.14 (strong effect) [42].

## 3. Results

### 3.1. Distribution of the Sample

The sample was 66.4% (*n* = 481) female and 33.6% (*n* = 243) male. Female veterinarians were significantly younger (p_Mann-Whitney_ < 0.002). The mean age of female veterinarians was 41.0 ± 9.72 years, and that of male veterinarians was 43.7 ± 10.79 years. Most veterinarians worked with small animals (55.7%; mean age 42.5 ± 10.16 years; 69.8% female), followed by large animals (16.9%; mean age 38.84 ± 9.58 years; 65.9% female) and a mix of small and large animals (15.3%; mean age 42.38 ± 11.05 years; 56.8% female). The mean age of veterinarians of laboratory (50.0% female) was 39.8 ± 9.17 years and the mean age of veterinarians of public administration (67.2% female) was 45.33 ± 9.76 years. Further socio-demographic and occupation-related data are shown in Table 2, including family status, which showed varying distributions among the groups, though the differences were not statistically significant (*p*_χ^2^_ = 0.159). Parental status revealed a significant difference (*p*_χ^2^_ = 0.009). The small group had a higher proportion of individuals with children (53.3%) compared to the other groups. In contrast, the proportion of individuals without children was highest in the group of large animals (21.1%).

The nature of the employment relationship varied significantly among the groups (*p*_χ^2^_ < 0.001). The majority of the small animals were self-employed (61.2%), whereas the pub adm predominantly consisted of employed individuals in public service (54.9%) and civil servants (83.3%). The field of activity also showed significant differences (*p*_χ^2^_ < 0.001). A large proportion of individuals in the group of small animals worked in big cities (>100,000 population) and medium and small towns.

**Figure 3:**
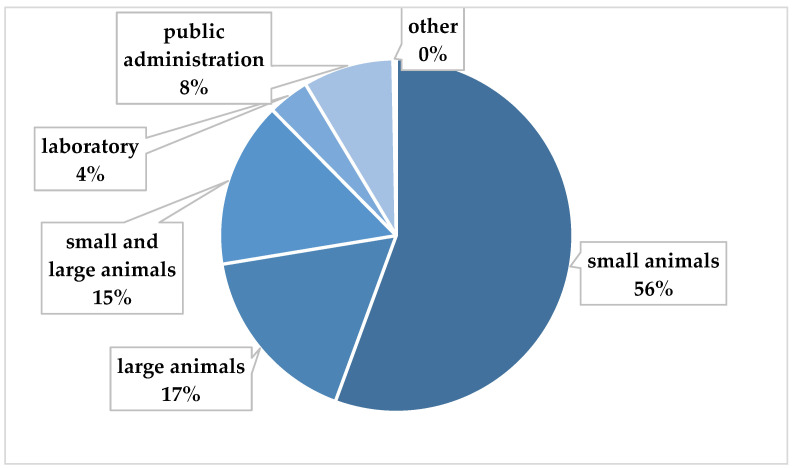
Distribution of veterinarians regarding main working field.

The distribution of veterinarians according to specialty is shown in Figure 3.

This categorization by working will be taken up again in the following sections.

### 3.2. Analysis of Overcommitment

**Figure 4:**
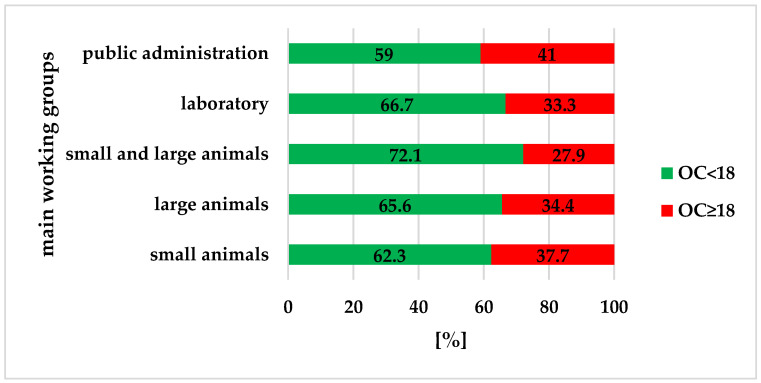
Frequencies of subjects with normal or excessive OC (*p*_χ_^2^ = 0.340).

The 75th percentile of the OC score was 18. Thus, the cutoff value for elevated OC was 18. Most veterinarians (65%) showed normal OC, and 35% of veterinarians showed an elevated OC; there were no significant group differences (*p*_χ^2^_ = 0.340). Public administrators had the highest percentage (41%) of veterinarians with elevated OC (Figure 4).

The OC scores of all groups were above the normal limit. The OC total score for those with OC scores < 18 was 14.3 ± 2.28 [95% CI: 14.10–14.52]. and that of individuals with OC scores ≥ 18 was 18.4 ± 1.43 [95% CI: 19.25–19.60]. Small animal veterinarians had the highest OC. The OC score differed according to veterinary specialty (*p*_KW_ = 0.019). The OC score of each specialty is shown in Table 3.

### 3.3. Work-Related Behavior and Experience Patterns (AVEM) and Dimensions

A total of 724 subjects could be assigned to AVEM patterns (pure, accentuated or tendential patterns). The distribution is presented in Table 4. There was no significant difference in AVEM patterns among veterinary working fields (p_Fisher_ = 0.188). Sixty-six percent of all veterinarians were assigned to AVEM risk pattern A or B.

A significant group difference was observed for only the AVEM dimension “tendency toward resignation in the face of failure” (*p*_KW_ = 0.004). The pairwise comparisons with Bonferroni correction are compared in Table 5. The values were predominantly in the normal range. For some AVEM dimensions, they were at the upper normal limit in all groups, such as “willingness to work until exhausted” and “tendency toward resignation in the face of failure”. The values for the AVEM dimension “proactive problem solving” were in the lower normal range for all groups.

### 3.4. Analysis of Irritation among Veterinarians

The values of cognitive irritation, emotional irritation, and total scores were in the upper normal range or above the normal range, and are shown in Table 6. Small- and large-animal veterinarians scored the highest on the irritation subscales and total scale. There were no group differences.

### 3.5. Nonparametric Correlations among AVEM Dimensions: Irritation Scores and Overcommitment

**Figure 5:**
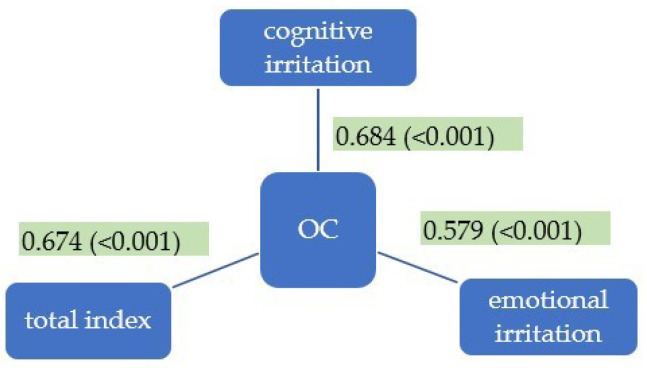
Correlations between overcommitment (OC) and subscales and total index of Irritation Scale.

Moderate positive correlations between OC and scores on cognitive irritation, emotional irritation, and the total scale were found (Figure 5). Both subscales of irritation (cognitive and emotional) correlate positively with overcommitment (ρ = 0.684, *p* < 0.001 resp. ρ = 0.579, *p* < 0.001).

Strong negative correlations between the AVEM dimension “distancing ability” and cognitive irritation scores (ρ = −0.837, *p* < 0.001) or total irritation scores (ρ = 0.723, *p* < 0.001) were also found. The other correlations are shown in Table 7.

### 3.6. General Linear Model (GLM)

Work group, gender, age, and OC were included in our model. When all of these factors are included, it can be seen that the IS scales and AVEM dimensions show significant differences between the groups studied. The model explains between 34 and 46% of the variance for the IS scales (R^2^ = 0.464 (cognitive IS), 0.339 (emotional IS), and 0.464 (total index). The constant terms, together, can also explain the variances for willingness to work until exhausted (R^2^ = 0.334), distancing ability (R^2^ = 0.455), and tendency to resignation in the face of failure (R^2^ = 0.214). Individually, gender and age showed no effect on the IS scales and AVEM dimensions in this model. OC showed a large effect (η^2^ > 0.14) on all IS scales and some AVEM dimensions (willingness to work until exhaustion, striving for perfection, distancing ability, tendency to resignation in the face of failure, and inner calm and balance). The results are shown in Table 8.

## 4. Discussion

The aim of this study was to investigate overcommitment, work-related behavior and experience, and psychological stress reactions in the form of irritation as a preliminary stage of stress before the onset of mental illness, and their correlations with overcommitment and AVEM patterns among German veterinarians in different working fields. Specifically, the study aimed to test the following hypotheses: Veterinarians in direct contact with animals (small animals, large animals) will show higher overcommitment, more risky work behavior patterns (A and B). and higher cognitive irritation and emotional irritation than veterinarians in other working areas (e.g., laboratory, public administration).

Overall, veterinarians working directly with small animals had the highest overcommitment scores, supporting the first hypothesis. Nearly two-thirds of all veterinarians conformed to AVEM risk patterns. Significant differences were observed in the dimension of tendency to resignation in the face of failure. Small-animal veterinarians had the highest score (6.2 ± 2.00), indicating a higher tendency toward resignation. Irritation and overcommitment scores were moderately to strongly correlated with AVEM dimensions and showed negative associations with the AVEM domain of emotions. Small- and large-animal veterinarians and those reporting an “other” specialty had the highest irritation scores. Overcommitment scores were moderately associated with irritation scores. Gender and working field had no influence of the results. To summarize the results, there were only partial differences in veterinary working fields, such as direct or indirect contact with animals. Thus, these results do not clearly indicate that veterinarians without direct contact with animals exhibit differences in OC, irritation, or other AVEM patterns or dimensions. For example, veterinarians in public administration (e.g., those responsible for impounding animals due to neglect) may potentially experience strong psychological distress, which could lead to increased commitment or overcommitment in their efforts to save these animals from misery.

OC can be viewed as an independent personality variable (trait) or as an outcome variable dependent on changes in the work environment (state) [43]. In this study, we took a trait perspective. The OC scores of German veterinarians ranged from 15.3 ± 3.20 (mixed animals) to 16.4 ± 3.11 (small animals). There were group differences between veterinarians who treated small and large animals and those in public administration, as well as between veterinarians who treated both large and small animals and those who treated small animals only. The veterinarians in this sample had higher OC scores than other German occupational groups, e.g., medical assistants (14.9 ± 3.5), nurses (14.5 ± 4.0), bank clerks (14.4 ± 3.9), and teachers (18.8 ± 3.9) [44]. The OC scores of Ukrainian anesthesiologists were 13.4 ± 2.7 in the total sample [45]. Studies have highlighted the negative consequences of overcommitment, such as burnout [46,47] and emotional exhaustion [32]. Silva et al. reported that overcommitment was associated with a higher prevalence of mild psychiatric disorders [48]. OC showed a high impact of the results of the Irritation Scale and AVEM dimensions (correlation analysis and GLM). Higher OC scores were associated with a worse quality of life, somatic symptoms, sleep problems, and a depressed or anxious mood [49].

The cognitive irritation score ranged from 13.3 ± 6.14 (laboratory) to 15.6 ± 4.69, and the emotional irritation score ranged from 17.3 ± 8.35 (mixed animals) to 19.3 ± 7.51 (large animals). These scores are at or above the upper limit of the norm. There were no significant group differences in irritation scores according to veterinary working field. Cognitive irritation scores in the general German population were 11.1 ± 4.3 points on average, and those in German educators were 11.7 ± 4.5 points [36]. Emotional irritation scores were 15.9 ± 5.7 points and 12.9 ± 5.4 points in the general German population and in hospital staff, respectively. International differences in irritation scores are not known. Cognitive irritation seems to be associated with workload, and high emotional irritation may indicate the presence of social stressors. Negative emotions do not stop at the end of work, often pervade leisure time and are unable to be relieved by normal recreational breaks. This represents an imbalance between personal resources and everyday stressors, which is a product of the interaction between individuals and the environment [36].

Sixty-six percent of veterinarians conformed to risky AVEM patterns (A or B), ranging from 54% (in mixed-animal veterinarians) to 70% (in small-animal veterinarians). These findings are supported by other studies that examined burnout symptoms in veterinarians. While some tentative comparisons may be drawn between the AVEM scores and burnout measures, it should be acknowledged that these tools conceptualize burnout differently, and thus only tentative claims may be made. Nevertheless, these data are interesting to mention. For example, using the Maslach Burnout Inventory (MBI), 493 out of 1.272 (38.8%) Canadian veterinarians were found to be affected by burnout [50], like the rates of Australian veterinarians who scored above the cutoff values in all three domains of the Copenhagen Burnout Inventory (CBI) (personal burnout: 37.0%; work burnout: 35.8%; client burnout: 24.8%) [16]. This underlines the need to prevent poor mental health in veterinary professions. Stable and sustained mental and physical health depend on various individual and general characteristics. These characteristics are mainly influenced by the profession and the associated stressors [51,52,53]. In the veterinary profession, working conditions such as night and weekend duties, lack of holidays, insufficient salary, overtime or deadline pressures also influence the job satisfaction and well-being of veterinarians [11].

More veterinarians conformed to AVEM risk patterns than individuals in other professions: 32% of prehospital emergency medical personnel [54], 40% of physicians [55], 47% of psychotherapy trainees [56], and 41% of nurses in the hospital setting [57]. AVEM risk patterns are also related to higher effort–reward imbalance (ERI), overcommitment, dissatisfaction, lower recovery, and anxiety [54,56,58]. AVEM dimension scores differed according to veterinary specialty. Since a high proportion of veterinarians exhibited AVEM risk patterns, it is not surprising that these rates did not differ significantly among working fields. In such cases, scores on individual dimensions play a role. These scores were predominantly within the normal range.

Because overcommitment scores were positively correlated with irritation scores, and AVEM scores in the area “emotions associated with work and with life in general” were negatively correlated with irritation and overcommitment scores, preventive behavioral measures are recommended. In particular, the correlation between overcommitment and irritation underscores the pressures veterinarians face due to excessive professional commitments. Overcommitment, as high intrinsic expenditure component, contributes to emotional exhaustion, rumination, and reduced job satisfaction [22,59,60]. Overcommitment can be exacerbated by inadequate compensation and prolonged stress [59,60], which are common in the veterinary profession. The decisive factor is whether the work and stress situation is subjectively experienced by a person as a challenge or as a (false) burden and how this affects their state of health and performance [61]. This excessive tendency to exert effort can increase health risks due to the effort–reward imbalance and be a health risk factor [62,63,64,65]. Economic pressures, such as lower income compared to human medical professionals and the financial burdens of running a practice, compound the stress veterinarians experience [66]. In addition, the organizational context (safety climate) plays a critical role [67]. The demanding nature of veterinary work, coupled with a lack of adequate support systems, creates an environment in which prolonged stress is prevalent [68]. This stress can lead to professional burnout, which manifests as chronic fatigue, cynicism, and a sense of reduced professional efficacy [3,68]. The high levels of irritability observed in our study participants reflect the emotional toll of these working conditions.

Addressing these issues requires a multifaceted approach. Improving compensation and creating family-friendly, flexible work arrangements can alleviate some of the economic and organizational pressures. In addition. implementing preventive measures such as regular mental health check-ups and stress management programs can help reduce the risk of burnout and depression. These measures should help veterinarians to distance themselves from their work. The ability to distance oneself from one’s work facilitates mental recovery from work. A low ability to distance oneself from work means that it is difficult to “switch off” from thinking about work, which hinders the ability to recover. Indeed, the ability to recover is well-known as an essential protective factor against mental illness [69]. Distancing ability was negatively correlated with irritation and overcommitment scores. Thus, it can be assumed that a higher distancing ability reduces irritation and overcommitment. Possible interventions include learning to say no to extra tasks, changing work organization and habits, and improving time management.

In summary, although small-animal veterinarians had higher overcommitment scores, the lack of significant differences in AVEM risk patterns and irritability scores across specialties suggests that risky work behaviors and irritability are pervasive across working fields. Thus, the hypothesis is only partially supported.

### 4.1. Strengths of the Work

A sample size of 724 is very good. The protocol was pre-registered [37]. The Irritation Scale has received little attention in the international literature. As described above, irritation represents a state between fatigue and clinical diagnosis, with “mild” symptomatology. This tool is particularly suitable for preventive use, as elevated values can signal the need to implement occupational health management measures. Further studies are needed in this area, because prevention and health promotion measures and their evidence should be adapted and evaluated in the setting of veterinary medicine. With the recording of OC, we can make assessments as to whether the occupational situation and characteristics of the work environment are subjectively experienced as a challenge or as a (mis)burden. It depends on these assessments as to whether and how this can have a corresponding effect on the state of health and performance. It is known from the literature that individuals who exhibit an excessive propensity to spend and a high effort–reward imbalance are more likely to report health impairments, for example cardiovascular disease and psychological complaints [31,62,63,70].

### 4.2. Limitations

The focus of this study was on veterinary working fields, so the age difference between the sexes was not further investigated. However, age is often associated with work experience; thus, this variable should be taken into account in the future. Additionally, the conclusions about this group should only applied with caution. Participation was voluntary. It is possible that veterinarians with very high perceived chronic stress reactions did not participate in the study. In addition, when interpreting the results, it should be noted that the information provided by the participating veterinarians depended on their current condition, mood and other influences, and that the results are therefore subject to a certain degree of fluctuation. The reliability of the individual AVEM dimension scores in German veterinarians was rated as only moderate. Nevertheless, the procedure is suitable for introducing targeted health management and promotion measures. There is a lot of discussion in the literature as to how irritation should be defined. This study claims that irritation is an indicator of work-related stress consequences and that it is a state between mental fatigue and mental illness, alongside the definition from Mohr et al. It can also be a natural short-lived reactive state, because the questionnaire itself does not specify a period of exposure.

The data from this study should be interpreted with caution due to the cross-sectional nature of the research, which limits the ability to establish causal relationships between psychological stress reactions, overwork, and work-related behaviors. Although significant associations were observed, these findings cannot determine the direction or causality of these relationships. Longitudinal studies are needed to clarify the temporal sequence of stress reactions and their contributing factors among veterinarians. In addition, the uneven distribution of veterinarians across practice settings and the reliance on self-report measures may also influence the results. Therefore, further research is needed to validate these findings and provide a clearer understanding of the causal mechanisms involved.

### 4.3. Implications for Occupational Medicine and Healthcare

The high prevalence of psychological stress reactions among German veterinarians requires urgent attention. Concrete measures are needed to improve working conditions and support mental health in the veterinary profession. Despite the uneven distribution of specialties, the findings highlight the pervasive nature of risky work behaviors and stress reactions across all veterinary specialties. The implementation of targeted interventions at both organizational and individual levels is essential to reduce over-involvement, enhance distancing skills, and promote healthy work patterns. Further research should focus on the development and evaluation of effective stress management interventions tailored specifically for veterinarians, taking into account the influence of gender, age and work experience on stress levels.

Research is needed to differentiate stressors according to the specific workplace conditions, e.g., in the context of risk assessments and the derivation of measures. Risk assessments. including mental risk assessments. are mandatory in Germany under the Occupational Health and Safety Act [71]. This applies to organizations with one or more employees. Workplace promotion of health should also be improved, e.g., by identifying organizational and personal resources. Organizational measures should be implemented according to the results of the risk assessment. Greater control by relevant public administration is needed to support the preparation of risk assessments and the development of corresponding measures.

However, it is unclear whether such improvements in occupational health and safety are feasible in veterinary practice. Working conditions and perceived burdens in veterinary medicine may not be easily changed, such as conflicts with pet owners and (unnecessary) euthanasia at the request of pet owners rather than for medical indication. In this case, the support of managers, team meetings or expert panels is recommended. Regarding reductions in work intensity, resource decisions in terms of staff or available time are recommended. These decisions should be adjusted according to the reasonable profitability of the organization. However, most resources for the development of protective measures (available time, structures) are lacking [72].

Personal resources can be strengthened through individual health management or health promotion measures. The AVEM applied here indicates opportunities for targeted interventions. Depending on individual dimension scores or AVEM patterns, suitable interventions can be implemented. For example, AVEM risk patterns can be altered through relaxation and compensation, training regarding satisfaction, the setting of work task-related goals, stress analysis and management, and the promotion of a team mentality. Individuals with AVEM pattern S could benefit from interventions that increase motivation as a protective function [29]. In the workplace setting, this could benefit the “subjective importance of work” or “work-related ambition” (e.g., in the form of regular training and knowledge enhancement). It should be noted that the experience of the AVEM pattern S is not necessarily related to personality alone, but can be an expression of a lack of occupational challenge [30].

Veterinary medicine is often portrayed in a negative light due to the high levels of stress, burnout, and mental health issues reported within the profession. While this negative view has some basis, it overlooks the fact that many veterinarians find great value and satisfaction in their work. The longstanding focus on challenges and problems within the profession has, of course, identified significant issues. However, many veterinarians enjoy their work and derive fulfillment from helping animals, building strong client relationships, and making meaningful contributions to public health.

Despite the prevalence of studies highlighting the difficulties, relatively little research has been conducted on the strengths and benefits of veterinary medicine. Recognizing both the challenges and the rewards can provide a more balanced perspective, and it is important to examine not only the stressors, but also the factors that lead to job satisfaction and commitment to the field. A comprehensive understanding of both the positive and negative aspects is valuable in improving the well-being of veterinary professionals.

## 5. Conclusions

Psychological stressors are high among veterinarians. Further studies are needed to investigate intervention measures in veterinary medicine settings to promote health.

Based on the results of this study and other studies, there is a need to improve the mental health of veterinarians. Attention should be given to raising awareness of mental health and the effects of overcommitment and (risky) work-related patterns of behavior and experience. Veterinarians should be informed about the risks and symptoms of stress reactions. This can include developing targeted stress management programs to help veterinarians cope with the stressors of their profession. These programs can include techniques such as relaxation exercises, time management and building social support. It is also important to improve working conditions in veterinary practice. Such improvements could include flexible working hours, adequate breaks, and support to cope with high workloads. Measures should be developed to identify veterinarians at an increased risk of mental distress at an early stage. Such individuals should be offered support in the form of counseling or therapy.

These measures can help to promote the mental health of veterinarians and to reduce the risk of stress reactions due to veterinary practice. It is important that both individual veterinarians and healthcare organizations and public administration work together to implement these recommendations and improve veterinarians’ well-being.

## Figures and Tables

**Table 1:** Score evaluation of Irritation Scale.

	Expression According to Score		
	Below Average	Average	Above Average
cognitive irritation	≤5	6–15	≥16
emotional irritation	≤8	9–20	≥21
IS total index	≤14	15–34	≥35

**Table 2:** Sociodemographic data of the study population.

Variables		Main Working Groups				
		Small	Large	Mixed	Lab	Pub Adm
Sex *n* (%)	Female	282 (58.6)	81 (16.8)	63 (13.1)	14 (2.9)	41 (8.5)
	Male	121 (49.8)	41 (16.9)	48 (19.8)	13 (5.3)	20 (8.2)
	*p*_χ_^2^ = 0.049					
Age	AV ± SD median (min–max) [95%CI]	42.49 ± 10.2 42 (24–72) [41.70–43.27]	38.84 ± 9.6 38 (25–79) [37.50–40.19]	42.38 ± 11.1 41 (25–70) [40.73–44.03]	39.77 ± 9.2 38 (25–58) [36.62–42.92]	45.33 ± 9.8 44.5 (28–68) [43.43–47.23]
	p_Bon_ large/mixed = 0.06; large/pub adm < 0.001; lab/pub adm = 0.025; small/pub adm = 0.005					
Family status (*n* (%))	Single	242 (56.7)	79 (18.5)	58 (13.6)	18 (4.2)	30 (7.0)
	Married	142 (55.3)	38 (14.8)	45 (17.5)	7 (2.7)	25 (9.7)
	Widowed	5 (44.4)	0	1 (11.1)	1 (11.1)	3 (33.3)
	Divorced	15 (48.4)	5 (16.1)	7 (22.6)	1 (3.1)	3 (9.7)
	*p*_χ^2^_ = 0.159					
Children (*n* (%))	Yes	130 (53.3)	32 (13.1)	45 (18.4)	9 (3.7)	28 (11.5)
	No	171 (57.4)	63 (21.1)	39 (13.1)	8 (2.7)	17 (5.7)
	*p*_χ^2^_ = 0.009					
Employment relationship (*n* (%))	self-employed	183 (61.2)	51 (17.1)	64 (21.4)	0	1 (0.3)
	employed (public service)	8 (11.3)	5 /7.0)	1 (1.4)	18 (25.4)	39 (54.9)
	Civil servant	1 (4.2)	2 (8.3)	0	1 (4.2)	20 (83.3)
	Private sector/industry	5 (26.3)	5 (26.3)	5 (15.8)	6 (31.6)	0
	Trainee/assistant doctor	120 (66.3)	37 (20.4)	24 (13.3)	0	0
	Others	8 (57.1)	1 (7.1)	4 (26.6)	0	1 (7.1)
	Doctoral candidates	2 (33.3)	2 (33.3)	0	2 (33.3)	0
	employed in practice/clinic	76 (69.1)	19 (17.3)	15 (13.6)	0	0
	*p*_χ^2^_ < 0.001					
Field of activity (*n*, (%))	Big City (>100,000)	134 (72.0)	10 (5.4)	4 (2.2)	16 (8.6)	22 (11.8)
	Medium and small town (<100,000)	182 (73.4)	14 (5.6)	25 (10.1)	9 (3.6)	18 (7.3)
	Rural	87 (30.0)	98 (33.8)	82 (28.3)	2 (0.7)	21 (7.2)
	*p*_χ^2^_ < 0.001					
Weekly working hours (only employed)	<20 h	20 (76.9)	3 (11.5)	2 (7.7)	0	1 (3.8)
	20–30 h	52 (62.7)	11 (13.3)	7 (8.4)	6 (7.2)	7 (8.4)
	30–40 h	67 (54.9)	17 (13.9)	8 (6.6)	8 (6.6)	22 (18.0)
	40–50 h	78 (45.6)	30 (17.5)	29 (17.0)	8 (4.7)	26 (15.2)
	>50 h	40 (48.2)	24 (28.9)	12 (14.5)	4 (4.8)	3 (3.6)
	*p*_χ^2^_ = 0.002					

Notes: small = small animals; large = large animals; mixed = small and large animals; lab = labor-atory; pub adm = public administration; AV ± SD = average and standard deviation; min = minimum; max = maximum; CI = confidence interval; *p*_Bonferroni_ = pairwise comparison with Bonferroni correction; *p_χ_*^2^ = Pearson Chi square test.

**Table 3:** Results of overcommitment regarding the main working fields.

Main Working Groups	OC Score (*n* = 724)	*p*_KW_	*p*_Bonferroni_
	AV ± SD; Median (Min–Max) [95%CI]		
small (*n* = 403)	16.4 ± 3.11; 17 (6–24) [16.13–16.74]	0.019	mixed/pub adm 0.042 small/mixed 0.001
large (*n* = 122)	16.0 ± 3.11; 16 (10–14) [15.48–16.59]		
mixed (*n* = 111)	15.3 ± 3.20; 15 (6–23) [14.69–15.89]		
lab (*n* = 27)	15.4 ± 3.59; 16 (6–22) [14.03–16.68]		
pub adm (*n* = 61)	16.3 ± 3.25; 17 (9–22) [15.46–17.13]		

Notes: small = small animals; large = large animals; mixed = small and large animals; lab = laboratory; pub adm = public administration; AV ± SD = average and standard deviation; min = minimum; max = maximum; CI = confidence interval; *p*_KW_ = Kruskal–Wallis test; *p*_Bonferroni_ = pairwise comparison with Bonferroni correction.

**Table 4:** Distribution of the veterinarians in the groups.

Main Working Groups	AVEM Risk Pattern		AVEM Pattern	
	A	B	G	S
	*n* (%)			
small (*n* = 407)	108 (26.8)	174 (43.2)	52 (12.9)	69 (17.1)
large (*n* = 123)	32 (26.2)	44 (36.1)	16 (13.1)	30 (24.6)
mixed (*n* = 111)	24 (21.6)	45 (32.4)	17 (15.3)	34 (30.6)
lab (*n* = 28)	6 (22.2)	11 (40.7)	2 (7.4)	8 (29.6)
pub adm (*n* = 61)	13 (21.3)	27 (44.3)	10 (16.4)	11 (18.0)
total (*n* = 724)	183 (25.3)	292 (40.3)	97 (13.4)	152 (21.0)

Notes: small = small animals; large = large animals; mixed = small and large animals; lab = laboratory; pub adm = public administration.

**Table 5:** Expressions of AVEM dimension of each main working group.

AVEM Dimensions	Main Working Group (*n* = 732)						
	Small	Large	Mixed	Lab	Pub Adm		
	AV ± SD median; (min–max) [95%CI]					*p*_KW_	*p*_Bonferroni_
Subjective importance of work	4.2 ± 2.09 4; (1–9) [4.01–4.42]	4.0 ± 2.08 4; (1–9) [3.65–4.42]	4.3 ± 2.17 4; (1–9) [3.91–4.72]	3.6 ± 2.39 3; (1–9) [2.61–4.50]	3.6 ± 2.08 3; (1–8) [3.09–4.16]	0.123	-
Work-related ambition	4.7 ± 2.28 5; (1–9) [4.48–4.93]	4.9 ± 2.26 4; (1–9) [4.50–5.31]	4.3 ± 2.17 4; (1–9) [3.89–4.71]	4.7 ±2.45 4; (1–9) [3.82–5.07]	4.4 ± 2.45 4; (1–9) [3.82–5.07]	0.234	-
Willingness to work until exhausted	5.9 ±2.29 6; (1–9) [5.71–6.16]	6.1 ± 2.10 6; (2–9) [5.75–6.50]	5.8 ± 2.35 6; (1–9) [5.35–6.24]	5.4 ±2.59 6; (1–9) [4.42–6.47]	5.7 ± 2.59 (1–9) [5.14–6.30]	0.747	-
Striving for perfection	5.3 ± 2.15 5; (1–9) [5.04–5.46]	4.7 ± 2.13 4; (1–9) [4.27–5.03]	4.8 ± 2.20 5; (1–9) [4.33–5.16]	5.0 ± 1.61 5; (1–7) [4.40–5.67]	5.1 ± 2.13 5; (1–9) [4.50–5.60]	0.051	-
Distancing ability	4.1 ± 2.12 4; (1–9) [3.86–4.28]	4.2 ± 2.13 4;(1–9) [3.82–4.59]	4.5 ± 2.26 4; (1–9) [4.09–4.94]	5.1 ±2.67 5; (1–9) [4.06–6.17]	4.6 ± 2.11 4; (1–8) [4.08–5.16]	0.071	-
Tendency to resignation in the face of failure	6.2 ± 2.00 7; (1–9) [6.01–6.40]	5.7 ± 2.01 6; (1–9) [5.36–6.08]	5.4 ± 2.26 5; (1–9) [5.01–5.86]	5.7 ± 1.84 6; (2–9) [4.94–6.39]	5.0 ± 1.92 6; (2–9) [5.21–6.20]	0.004	small/mixed 0.001 small/pub adm 0.045 small/large 0.022
Proactive problem-solving	3.6 ± 2.00 3; (1–9) [3.42–3.81]	4.0 ± 1.80 4; (1–9) [3.76–4.49]	4.1 ± 1.93 4; (1–9) [3.76–4.49]	3.9 ± 2.08 5; (1–8) [3.07–4.71]	3.6 ± 1.83 4; (1–8) [3.14–4.07]	0.058	-
Inner calm and balance	4.7 ± 1.92 5; (1–9) [4.56–4.93]	5.0 ± 2.02 5; (1–9) [4.65–5.37]	5.1 ± 2.02 5; (1–9) [4.71–5.47]	4.3 ± 1.94 4; (1–9) [3.57–5.10]	4.8–1.91 5; (1–9) [4.28–5.26]	0.203	-
Experience of success at work	4.4 ± 2.47 4; (1–9) [4.11–4.60]	4.5 ± 2.43 4; (1–9) [4.06–4.93]	4.9 ± 2.21 5; (1–9) [4.45–5.28]	5.0 ± 2.32 5; (1–9) [4.12–5.96]	4.3 ± 2.24 4; (1–8) [3.75–4.90]	0.158	-
Satisfaction with life	4.0 ± 2.36 4; (1–9) [3.79–4.25]	4.1 ± 2.26 4; (1–9) [3.69–4.50]	4.6 ± 2.33 5; (1–9) [4.12–5.00]	4.0 ± 1.98 4; (1–9) [3.22–4.78]	3.8 ± 2.03 4; (1–9) [3.32–4.36]	0.193	-
Experience of social support	4.4 ± 2.09 5; (1–9) [4.18–4.59]	4.3 ± 1.95 4; (1–9) [3.92–4.62]	4.8 ± 2.02 5; (1–9) [4.40–5.16]	3.9 ± 1.94 4; (1–9) [3.16–4.69]	4.1 ± 1.96 5; (1–9) [3.63–4.63]	0.083	-

Notes: small = small animals; large = large animals; mixed = small and large animals; lab = laboratory; pub adm = public administration; AV ± SD = average and standard deviation; min = minimum; max = maximum; CI = confidence interval; *p*_KW_= Kruskal–Wallis test.

**Table 6:** Results of Irritation Scale regarding main working fields.

Main Working Groups	Irritation Scale		
	Cognitive	Emotional	Index
	AV ± SD; Median (Min–Max) [95%CI]		
small (*n* = 403)	15.6 ± 4.69; 17 (3–21) [15.13–16.04]	19.7 ± 7.95; 20 (5–35) [18.92–20.48]	35.3 ± 11.49; 36 (8–56) [34.15–36.40]
large (*n* = 122)	15.1 ± 4.78; 16 (3–21) [14.20–15.92]	19.93 ± 7.51; 19 (5–35) [18.59–21.28]	35.0 ± 11.14; 35 (11–56) [33.00–36.99]
mixed (*n* = 111)	14.2 ± 5.65; 15 (3–21) [13.10–15.22]	17.3 ± 8.35;18 (5–34) [15.73–18.87]	31.5 ± 13.11; 34 (8–55) [28.99–33.93]
lab (*n* = 27)	13.3 ± 6.14; 15 (4–21) [10.83–15.69]	18.0 ± 9.99; 15 (5–35) [14.08–21.99]	30.3 ± 14.48; 30 (9–54) [25.57–37.03]
pub adm (*n* = 61)	14.4 ± 5.01;15 (5–21) [13.09–15.66]	19.2 ± 7.29; 19 (5–35) [17.30–21.03]	33.5 ± 10.78; 35 (11–56) [30.78–36.30]
*p*_KW_	0.071	0.070	0.060

Notes: small = small animals; large = large animals; mixed = small and large animals; lab = laboratory; pub adm = public administration; AV ± SD = average and standard deviation; min = minimum; max = maximum; CI = confidence interval; *p*_KW_ = Kruskal–Wallis test; the *p*_KW_ was above 0.05. Therefore, the Bonferroni correction was not applied. Yellow background means overshot values for this area.

**Table 7:** Results of Irritation Scale regarding AVEM dimensions.

AVEM dimensions	Irritation Scale			ERI OC
	Cognitive	Emotional	Total Index	OC Score
Subjective importance of work	0.130 <0.001	0.028 0.458	0.073 0.048	0.154 <0.001
Work-related ambition	0.162 <0.001	0.062 0.095	0.105 0.005	0.183 <0.001
Willingness to work until exhausted	0.484 <0.001	0.414 <0.001	0.474 <0.001	0.566 <0.001
Striving for perfection	0.405 <0.001	0.325 <0.001	0.382 <0.001	0.437 <0.001
Distancing ability	−0.837 <0.001	−0.566 <0.001	−0.723 <0.001	−0.679 <0.001
Tendency to resignation in the face of failure	0.517 <0.001	0.561 <0.001	0.588 <0.001	0.428 <0.001
Proactive problem-solving	−0.159 <0.001	−0.307 <0.001	−0.279 <0.001	−0.143 <0.001
Inner calm and balance	−0.449 <0.001	−0.630 <0.001	−0.620 <0.001	−0.433 <0.001
Experience of success at work	−0.231 <0.001	−0.312 <0.001	−0.312 <0.001	−0.150 <0.001
Satisfaction with life	−0.416 <0.001	−0.508 <0.001	−0.518 <0.001	−0.354 <0.001
Experience of social support	−0.240 <0.001	−0.305 <0.001	−0.311 <0.001	−0.219 <0.001

Notes: weak correlation, 0.100 to 0.399; moderate correlation, 0.400 to 0.699; and strong correlation ≥ 0.700; green = positive correlation; red = negative correlations. The more intense the color, the stronger the correlation.

**Table 8:** Results of the general linear model analysis.

	Corrig. Model			Sex		Age		OC-Score		Working Group	
	F	*p*	η^2^	*p*	η^2^	*p*	η^2^	*p*	η^2^	*p*	η^2^
Cognitive scale	90.315	<0.001	**0.469**	0.312	0.001	0.007	0.010	<0.001	**0.444**	0.219	0.008
Emotional scale	53.962	<0.001	**0.345**	0.940	0.000	0.122	0.003	<0.001	**0.327**	0.620	0.004
Irritation total index	90.266	<0.001	**0.469**	0.630	0.000	0.021	0.007	<0.001	**0.447**	0.607	0.004
Subjective importance of work	5.827	<0.001	0.054	0.658	0.000	0.001	0.015	<0.001	0.035	0.064	0.012
Work-related ambition	10.440	<0.001	0.093	0.138	0.003	<0.001	0.051	<0.001	0.029	0.890	0.002
Willingness to work until exhausted	52.847	<0.001	**0.341**	0.936	0.000	0.425	0.001	<0.001	**0.336**	0.135	0.010
Striving for perfection	24.963	<0.001	**0.196**	0.805	0.000	0.197	0.002	<0.001	**0.177**	0.149	0.009
Distancing ability	87.322	<0.001	**0.461**	0.626	0.000	0.031	0.006	<0.001	**0.440**	0.088	0.011
Tendency to resignation in the face of failure	29.139	<0.001	**0.222**	0.011	0.009	<0.001	0.025	<0.001	**0.160**	0.086	0.111
Proactive problem-solving	4.333	<0.001	0.041	0.953	0.000	0.003	0.012	<0.001	0.015	0.168	0.009
Inner calm and balance	24.471	<0.001	**0.193**	0.427	0.001	0.024	0.007	<0.001	**0.171**	0.248	0.008
Experience of success at work	4.965	<0.001	0.046	0.257	0.002	<0.001	0.017	0.001	0.014	0.423	0.005
Satisfaction with life	16.220	<0.001	**0.137**	0.340	0.001	0.108	0.004	<0.001	0.119	0.642	0.004
Experience of social support	8.239	<0.001	0.075	0.491	0.001	0.002	0.014	<0.001	0.058	0.143	0.010

Notes: weak effect size η^2^ < 0.06, moderate effect size η^2^ = 0.06–0.14, strong weak effect size η^2^ > 0.14. Bold markings mean strong effects.

## Data Availability

There are no plans to grant access to the full protocol, participant-level datasets, or statistical codes, as data contain potentially identifying information.
